# The rotation of medical staff in July 2020 post COVID-19

**DOI:** 10.1017/ipm.2020.80

**Published:** 2020-06-25

**Authors:** M. Cosgrave

**Affiliations:** Health Service Executive, North Dublin Mental Health Service, Dublin, Ireland; Royal College of Surgeons in Ireland, Department of Psychiatry, Dublin, Ireland

Dear Editor,

As we reach the mid-summer of 2020, it is usual in Irish medical circles to anticipate the largest annual changeover of medical staff in our hospitals and services. Some of the doctors leaving us will be taking stories and experiences with them from our services, and others will be coming to our services with unique narratives of their current postings. Some of our staff will be coming from or returning to other parts of the world for a new chapter in their careers.

In Psychiatry, the non-consultant hospital doctors met with many challenges during the initial surge of COVID-19 and were at the frontline in acute psychiatric units, emergency departments, general hospital wards, long-stay units, and in the community. They have spent many hours on call, often with no contact with medical colleagues from their own discipline. Some doctors were redeployed outside Psychiatry: a transfer of responsibility and exposure that will have brought its own particular challenges.

There have been further challenges in adhering to training standards among junior doctors. Competency-based training requirements have been difficult to maintain within psychiatry due to the widespread disruption of normal business.

Further challenges have included conducting acute emergency psychiatric assessments while trying to maintain social distancing. Sometimes, this may have been conducted by telephone or video consultation. There is an increasing consensus that remote consultation in acute presentations may add to the complexity of the assessment which may have put frontline clinicians under increased stress.

The COVID-19 pandemic will have affected us all but may have had a profound effect on our younger frontline staff in many ways, from experiences at work, disruption to training, personal illness or quarantine, separation from family, and the effects of the emergency on their loved ones.

Going forward, it is important for us all in medicine to be accessible to those who leave our services as well as those who remain within our services and that we are open to listening and providing support to all. Attention is required to the individual doctor’s training history and to their training requirements going forward.

Psychiatry, in particular, can take a lead on this as we have structures already in place as part of training such as Balint groups. Our day to day work is also closely related. The very structured training system in psychiatry might also be used to help structure the ongoing development of doctors within and outside training schemes across all medical specialties.

As a profession of Psychiatrists, we can be leaders in supporting junior doctors both locally and nationally.

Yours sincerely

Mary Cosgrave

